# Florid Proliferation of Hyalinized Vessels in a Spermatic Cord STAT6 Positive Solitary Fibrous Tumor and Its Potential Clinical Implications

**DOI:** 10.1155/2018/7462032

**Published:** 2018-06-28

**Authors:** Christopher P. Marquez, Haiyan Zhang, Jason Goodrum, J. Nicholas Sreshta, Marjan Afrouzian

**Affiliations:** ^1^Department of Pathology, University of Texas Medical Branch, Galveston, TX, USA; ^2^Department of Surgery, Division of Urology, University of Texas Medical Branch, Galveston, TX, USA

## Abstract

A solitary fibrous tumor (SFT) arising in the paratesticular region is a rare event. Typically most SFTs present as a lung mass and have a characteristic microscopic appearance. Although uncommon, SFTs may present at just about any anatomical site. Here we present a case of a SFT arising along the right spermatic cord, with histologic features mimicking a cellular angiofibroma. We describe the diagnostic immunohistochemical markers useful for arriving at its diagnosis. We also summarize our current understanding of the structural and molecular features that make up SFTs and discuss how these features may help us better understand the pathophysiology of pluripotent mesenchymal stem cell differentiation.

## 1. Introduction

SFTs typically present as a lung mass arising from the visceral pleura; however, since its description in 1931, extrapleural tumors have been identified at various other anatomical locations [1]. SFTs can present at any age, and while pleural SFTs most often occur during the 6^th^ and 7^th^ decades of life, extrapleural SFTs tend to manifest in adults aged 20 to 70 years old. SFTs have no sex predilection, except for the extrapleural fat-forming SFT variant, which is seen slightly more often in males than females. SFTs usually are benign and slow-growing; however, 10% of patients will have tumors that behave aggressively. To date, SFTs have not been linked to any etiologic agent [2, 3]. A solitary fibrous tumor presenting in the paratesticular region is unusual. To the best of our knowledge, at least 21 cases are reported in the literature, of which 9 were specifically noted to have arisen along the spermatic cord (Table 1).

Herein we report a case of a SFT arising along the right spermatic cord, with features mimicking a cellular angiofibroma. The diagnosis of SFT was ultimately confirmed by nuclear expression of STAT6 in the tumoral cells.

## 2. Case

**Figure 1:**
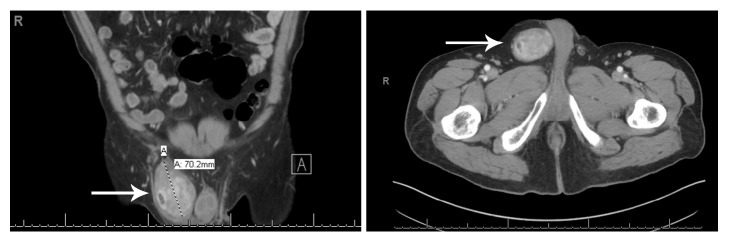
Computed tomography (CT) scans revealing a partially visualized, heterogenous enhancing right inguinal mass (arrows).

A 48-year-old male presented in surgery clinic with a clinical history of benign prostatic hyperplasia and a 6-year history of an enlarging right inguinal hernia, with associated increase in discomfort. On physical examination, a cystic mass was palpated on the superior right testicle, and a firm, solid mass was found in the right groin. The testicular mass was fully mobile within the subcutaneous space and minimally tender and did not appear to be connected to the external ring. The patient had no other complaints, and the rest of his physical examination was unremarkable. A follow-up computed tomography (CT) scan revealed a partially visualized, heterogenous, and enhancing right inguinal mass, raising the concern for a peripheral nerve sheath tumor or sarcoma of the spermatic cord (Figure 1).

**Figure 2:**
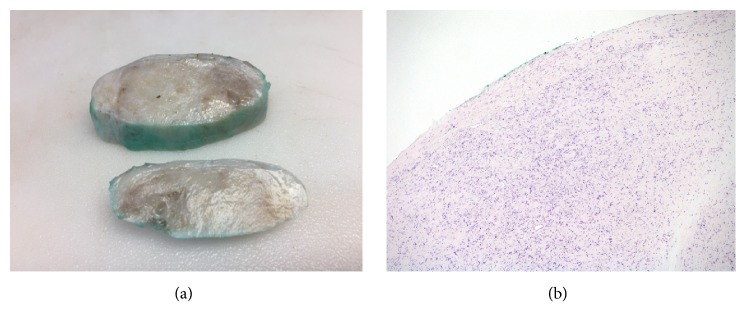
(a) Gross photograph of the tumor. The tumor is well-circumscribed with a thin fibrous membrane. (b) The tumor at low magnification, demonstrating the tumor border (H&E, x40).

The mass was surgically excised from the spermatic cord. During surgery, it was noted that the mass was located inside the external cord, but outside of the internal spermatic fascia. It had eroded through the aponeurosis of the external oblique muscle. Nonetheless, the mass could easily be separated from the spermatic cord and was submitted to pathology for evaluation. Macroscopically, the mass weighed 67.5 grams and measured 7 x 5.5 x 2.5 centimeters. Its outer surface was smooth, pink/white in color and covered by a thin membrane. The cut surface of the mass was white and firm and had a whorled texture containing occasional small cysts (Figure 2(a)). The tumor border is well delineated. The margin is inked green (Figure 2(b)).

**Figure 3:**
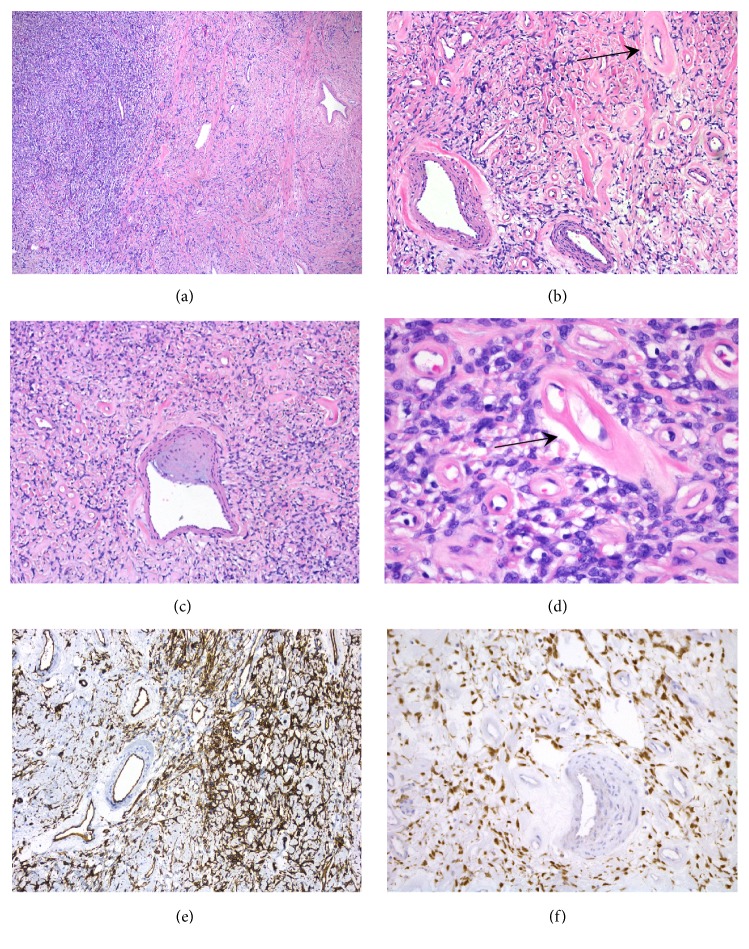
Microscopic features and immunohistochemical results. (a) Typical hypo- and hypercellular areas of the tumor (H&E, x40). (b) Vessels containing proliferating myocytes associated with complete mural hyalinization (arrow) (H&E, x100). (c) A vessel with mural myxoid degeneration (H&E, x100). (d) Bland looking neoplastic cells with oval to fusiform nuclei and little cytoplasm around hyalinized small vessels (arrow) (H&E, x400). (e) Numerous hemangiopericytoma-like vascular channels (CD34, x100). (f) STAT6 immunostain showing strong diffuse nuclear positivity in the neoplastic cells. The vascular myocytes are negative (STAT6, x200).

Microscopically, the tumor had a heterogeneous pattern-less architecture with alternating hypocellular and hypercellular areas, interstitial hyalinization, and intermixed with ropy collagen bands (Figure 3(a)). The most prominent feature of the tumor was found within the vascular compartment. Numerous small- to medium-sized vessels were present and showed a continuum of changes, ranging from thick-walled vessels showing proliferation of myocytes, to vessels revealing intimal thickening, to vessels with circumferential prominent mural hyalinization (Figure 3(b)). Some of the larger vessels displayed subendothelial mucoid degeneration resembling vascular changes observed during accelerated hypertension (Figure 3(c)). When the course of one of the vessels was followed, the vessel was found to have cellular myocytic proliferation in some segments followed by hyalinization in other segments. Occasional individual exuberant hemangiopericytoma-like vascular channels were also present (Figure 3(d)).

The neoplastic cells were ovoid to spindle-shaped with scanty pale cytoplasm and oval to fusiform nuclei (Figure 3(e)). Mitosis was scarce and at less than one per 10 high power fields, and the margins were free of tumor. Immunohistochemical results for the patient are listed in Table 2. The tumoral cells expressed CD34, BCL-2, and CD99. They were also focally positive for Estrogen Receptor (ER) and Progesterone Receptor (PR) and negative for Smooth Muscle Actin (SMA) and S100. A STAT6 stain was ordered and revealed nuclear positivity in the tumoral cells but negative staining in the vascular myocytes (Figure 3(f)). No follow-up data is available due to the recent removal of the tumor at the time of writing of this case report.

## 3. Discussion

Initially SFTs were believed to be tumors of pericytic origin; however, over the years and through ultrastructural studies, it was discovered that SFTs are tumors with neoplastic cellular heterogeneity consisting of undifferentiated perivascular cells, fibroblasts, myofibroblasts, pericytes, smooth muscle cells, and endothelial cells, all possibly arising from adult mesenchymal stem cells [1, 29, 30]. The relative proportion of each component within the tumor may be different from one SFT to the next [30]. SFT's anatomical presentation and characteristic architectural features usually help to distinguish it from other tumors. The tumor can vary greatly in appearance, depending on the relative proportion of tumoral cells to the surrounding fibrous stroma, with the classical hemangiopericytoma being at the cellular end of the spectrum, and the hyalinized form at the other end of the spectrum, representing the classical solitary fibrous tumor [1]. Characteristically, SFTs have a pattern-less architecture, with alternating hypercellular and hypocellular areas separated by bands of hyaline, and numerous thin-walled branching staghorn vessels. The neoplastic cells are typically ovoid to spindle-shaped, with limited pale cytoplasm, and scarce mitotic figures. SFTs may resemble benign neural or smooth muscle tumors. Our tumor is distinct from the typical presentation of SFTs, by its formation along the spermatic cord and morphological similarity to a cellular angiofibroma; moreover, the sequences of changes starting with myocyte proliferation, transmural myxoid changes, and then partial to complete circumferential hyalinosis were unique vascular features.

The presentation along the spermatic cord in our patient had led to a misdiagnosis of inguinal hernia for several years, thus delaying tumor removal. Therefore, even though spermatic cord SFT is a rare tumor, it should be considered in the differential diagnosis of spermatic cord masses, by the clinician. Another benign mesenchymal tumor in the urogenital region that can be confused with a spermatic cord SFT is cellular angiofibroma which is typically seen in the superficial soft tissue of the genital region, with the inguinal region being the most common site in men [26]. Histologically, our tumor had some important features of a cellular angiofibroma, notably presence of florid proliferation of minute-small- to medium-sized vessels with hyalinized walls.

The tumoral cells expressed CD34, ER, and PR, but not SMA or S100. This phenotype initially supported a diagnosis of cellular angiofibroma; however, additional staining of the spindle cells demonstrated CD99 and BCL-2 expression, raising the suspicion for SFT. Ultimately, the strong diffuse positive nuclear staining for STAT6 in the neoplastic cells established a diagnosis of spermatic cord SFT [31, 32]. If our case had been negative for STAT6, as is seen in a subset of SFT cases [3], then additional staining for desmin and FISH testing for* RB1* and* FOXO1* loci on chromosome 13q14 may be helpful [26]. Desmin is usually negative in SFTs [1] but may be positive in about 8% of cellular angiofibromas [24]. The deletion of the* RB1* and* FOXO1* loci would also support a diagnosis of cellular angiofibroma.

Of note, another important diagnostic consideration along the spermatic cord is the possibility of a dedifferentiated liposarcoma (DDLPS). Although rare, more cases have been reported of DDLPS along the spermatic cord than SFT [33]. However, there are several histologic clues that argue against a DDLPS in our case. It has also been shown that about 11% of DDLPS may show STAT6 expression secondary to amplification. In a study by Doyle et al., a total of 4 out of 35 cases (11%) examined showed STAT6 expression. Three showed moderate-to-strong multifocal staining and one with weak focal staining [34]. This is in contrast to our case, which showed strong diffuse positive nuclear staining. SFTs typically show diffuse nuclear staining (> 90% of cases), whereas cytoplasmic, not nuclear, staining is typically seen in > 95% of non-SFT cases expressing STAT6 [35]. Another typical feature seen in DDLPS, and not seen in our case, includes an architectural transition between the dedifferentiated component (increased mitosis and atypia) and the well-differentiated component of liposarcoma. Additionally, DDLPS tend to be of larger size, multinodular, and necrotic in the dedifferentiated component [36]. Nonetheless, if clinically suspected, it would be necessary to exclude DDLPS with MDM2 and CDK4 immunohistochemistry.

The vessels themselves did not share the same immunohistochemical pattern as the tumoral cells but expressed the staining patterns typically seen in normal vessels including CD34 positivity and STAT6 negativity. It may well be possible that the vascular myocytic proliferation seen in our case represents an angiogenic response to mitogens produced by the tumor, such as platelet-derived growth factor (PDGF) [30]. In 2013, Robinson and collogues demonstrated that PDGF-D, a potent transforming and angiogenic growth factor [37], showed significantly higher levels in SFTs compared to other tumor types. They postulated that the fusion of NAB2 gene with STAT6 gene, converted NAB2 from an early growth response 1 (EGR-1) repressor to an EGR-1 activator and that this genetic fusion is the driving factor in the development of SFTs [38]. The authors of this article believe that developing animal models of SFT through genetic engineering and induction of NAB2-STAT 6 fusion gene can be the next step in the research and development of treatment options for diseases linked to fibroblastic proliferation such as systemic sclerosis, interstitial lung disease, pulmonary hypertension, and even arteriosclerosis and malignant hypertension.

SFT is a great example of how next-generation sequencing has opened the doors of our understanding regarding tumor initiation and development over time. As we continue to learn more about this uncommon but interesting tumor, we may find that this tumor yet holds secrets to the pathophysiology of pluripotent mesenchymal stem cell differentiation.

## Figures and Tables

**Table 1:** Summary of cases.

References	Age	Greatest dimension (cm)	Region	Laterality
Fisher C, et al. [4]	46	10	Spermatic Cord	NS
Gold JS, et al. [5]	NS	NS	Spermatic Cord	NS
Xambre L, et al. [6]	67	10	Spermatic Cord	R
Honeck P, et al. [7]	64	4.3	Spermatic Cord	Left
Ilica AT, et al. [8]	20	14	Spermatic Cord	Right
Arrabal-Polo MA, et al. [9]	44	5	Spermatic Cord	Left
Topsakal K, et al. [10]	21	5	Spermatic Cord	Left
Barazani Y, et al. [11]	26	6.2	Spermatic Cord	Left
Hu S, et al. [12]	31	3	Spermatic Cord	Left
Jones MA, et al. (2 cases) [13]	NS	NS	Scrotum	NS
	NS	NS	Scrotum	NS
Shim JW, et al. [14]	38	11	Scrotum	Left
Márquez Moreno AJ, et al. [15]	67	9	Scrotum	Left
Ch Tsili A, et al. [16]	65	1.9	Scrotum	Left
García Torrelles M, et al. [17]	22	2.5	Scrotum	Left
Varela R, et al. [18]	64	20	Scrotum	Right
Lee GE, et al. [19]	61	5	Scrotum	Left
Parikh BJ, et al. [20]	42	8	Scrotum	Left
Zhou Y, et al. [21]	61	5.1	Scrotum	Left
Zhao X, et al. [22]	77	11	Scrotum	Right
Gutierrez-Diaz CM, et al. [23]	53	NS	Paratesticular	NS

NS: not specified.

**Table 2:** Comparison of the immunohistochemical profile of classical cellular angiofibroma, SFT, and our patient's spermatic cord tumor.

Immunostain	Cellular Angiofibroma	SFT	Patient's Tumor
CD34 [1, 2]	+ (60%)	+ (80-95 %)	+
ER [24, 25]	+ (35%)	–	+ (focal)
PR [24, 25]	+ (55%)	+ (patchy)	+ (focal)
SMA [1, 24]	+ (21%)	+ (20%)	–
S100 [1, 2]	–	– (usually)	–
CD99 [26, 27]	+ (2 of 4 cases reported)	+ (70-94 %)	+
bcl2 [1, 26]	Not reported	+ (30-96 %)	+
STAT6 [3, 28]	–	+ (>95%)	+ (Figure 3(f))
